# The Development and Validation of the Psychological Needs of Cancer Patients Scale

**DOI:** 10.3389/fpsyg.2021.658989

**Published:** 2021-06-03

**Authors:** Yao Chen, Fangyan Lin, Bo Wang, Yung-lung Tang, Jun Li, Lin Xiong

**Affiliations:** ^1^Radiation Therapy Center, Chongqing University Cancer Hospital, Chongqing, China; ^2^Faculty of Psychology, Southwest University, Chongqing, China; ^3^Department of Psychology, Chongqing University Cancer Hospital, Chongqing, China; ^4^Department of Urologic Oncology Surgery, Chongqing University Cancer Hospital, Chongqing, China

**Keywords:** psychological needs, cancer patients, scale development, palliative care, hospice care, mental health

## Abstract

In the present research, the Psychological Needs of Cancer Patients Scale (PNCPS) was developed and validated. Based on Group 1 (400 cancer patients), the exploratory factor analysis identified a 23-item scale with six factors: value and esteem (five items, i.e., reconsider the meaning and purpose of life), independence and control (six items, i.e., private space), mental car (three items, i.e., vent negative emotions), disease care (three items, i.e., acquire knowledge about disease), belonging and companionship (three items, i.e., spend more time at home), and security (three items, i.e., living conditions be better). The structure identified with Group 1 was further tested, based on Group 2 (199 cancer patients), for reliability and validity. The results showed that PNCPS has a clear factor structure and good psychometric characteristics. By taking into account the cultural background of Chinese patients, this scale will advance the study of the psychological needs of those with malignant tumors and thus has a certain reference value for other countries.

## Introduction

According to World Health Statistics 2020, cancer caused 9 million deaths in 2016 (WHO, 2020). In 2018, cancer was responsible for an estimated 9.6 million deaths. And according to the latest statistics from China’s National Cancer Center, the estimated incidence of malignant tumors nationwide was 285.83 per 100,000 in 2015. Malignant tumor patients form a large patient group. Importantly, the negative impact of cancer on patients is not only reflected in their physical symptoms but also reflected through social adjustment disorders and psychological discomfort (Peng et al., 2019). Although the reported prevalence of depression varies significantly (Massie, 2004; Pirl, 2004), the majority of rates for major depressive disorder fall between 10 and 25% of patients with malignant cancer tumors, while for probable cases of depression, the majority of reports fall into the 7–21% range (Pirl, 2004). Similarly, the reported prevalence of anxiety problems in cancer patients is higher than in others without cancer (Stark and House, 2000). Additionally, the fear of recurrence and post-traumatic stress disorder are two common psychological concerns among cancer survivors (Claire et al., 1997; Kangas et al., 2002; Strong et al., 2008).

Therefore, it is likely that many psychological needs of malignant tumor patients are not met. Need refers to a lack and imbalance in the human tissue system. In the present research, we aimed to develop a reliable and valid measure of the psychological needs of malignant tumor patients, appropriate for use in the Chinese cultural context.

The development of such a measure is important. First of all, investigating and meeting the psychological needs of patients with malignant tumors are conducive to improving their quality of life as part of palliative care and hospice care. With the development and progress of modern medicine, the survival time for malignant tumor patients is prolonged, and the treatment of cancer has become a long and painful process. From the moment of diagnosis, palliative care improves the quality of life of patients and their families facing physical, psychological, social, and spiritual challenges related to life-threatening disease and is an essential component of cancer care (WHO, 2021). An estimated 40 million people need palliative care each year, yet unfortunately, only about 14% of those in need worldwide are currently receiving it (WHO, 2020).

At the end of a patient’s life, hospice care provides comprehensive and active whole-person care for the patient and his/her family members. By providing psychological care and comfort, eliminating the fear of death, and paying attention to the patient’s comfort, the dying can face death calmly and leave safely (Chen, 2003). A survey and analysis of end-of-life care needs of patients with advanced cancer found that patients had high levels of physical (e.g., sleep and pain) and psychological (e.g., anxiety and depression) needs, and moderate levels of spiritual (e.g., death education) and social support (e.g., family contact) needs (Zhao et al., 2015). Treatment currently focuses on cure and therefore does not acknowledge the other but very real needs of the majority of patients who will die from their disease (Hilden et al., 2001). Under the cancer resource allocation scheme recommended by the WHO for developing countries, more resources should be devoted to pain control and end-of-life care for patients with advanced cancer than to cancer treatment itself (Coyne, 1997).

Investigating and meeting the psychological needs of patients with malignant tumors will help promote their mental health, as people with cancer often have mental health problems. Most current research focuses on reducing or preventing the suffering and negative reactions experienced by cancer survivors, thus promoting their mental health. For example, Strong et al. (2008) conducted a psychological intervention on cancer patients with depression and found that depression, anxiety, and fatigue in patients were improved. However, it is important to note that psychological health in cancer survivors is determined by both the presence or absence of distress as well as the presence or absence of a variety of positive psychological responses often subsumed under the concept of “posttraumatic growth” (Cordova et al., 2001; Cordova and Andrykowski, 2003; Stanton et al., 2006). However, very little research has looked at whether and, if so, how positive psychological responses could be fostered in cancer survivors. Research into the psychological needs of cancer patients could help compensate for this. In other words, while focusing on unmet needs, we can also focus on well-met needs and tap into the positive reactions of cancer survivors to promote their mental health.

A better understanding of the psychological needs of patients with malignant tumors can also contribute to improving their physical health. Both the occurrence and development of malignant tumors are closely related to the psychological state of patients. A large number of clinical studies and epidemiological investigations have found that some specific psychosocial factors, such as stress and depression, are risk factors for the occurrence and development of tumors. Thus, people with poor mental health are more likely to develop tumors, demonstrating that psychosocial factors are closely related to the occurrence, development, and prognosis of tumors (Price et al., 2001). Many researchers have found that psychological interventions for patients with malignant tumors can also have a positive impact on the patient’s condition. The research of Guo et al. (2019) showed that a personalized psychological intervention can effectively improve anxiety, depression, and negative mood in patients with lung cancer and improve their quality of life, satisfaction, and medication compliance. In addition to the above factors, psychological needs also include the patient’s need to understand medical information and medical services, which are directly related to treatment planning and treatment effects.

The satisfaction of psychological needs over the course of cancer treatment will affect treatment satisfaction and treatment effects (Walker et al., 2003). However, the current health-care systems worldwide are unable to meet the various psychological needs of patients (Walker et al., 2003; Marijnen et al., 2005; Cox et al., 2006), and patients are less satisfied with how their psychological needs are met by doctors than they are with the diagnosis and treatment of cancer.

In 2000, Billie Bonevski published the first questionnaire to assess the psychological needs of cancer patients (Bonevski et al., 2000). Since then, a range of tools have been developed to assess the needs of patients with cancer. The purpose and population of these studies vary a lot: some were proposed as specific to advanced stage of disease, clinical setting, or survivors, and some were targeted particular diagnoses (e.g., lung cancers) (Duke et al., 2003; Hodgkinson et al., 2007; Ahmed et al., 2011; Richards et al., 2011). Among then, a number of instruments were developed to assess multiple needs of general population, such as the Supportive Care Needs Survey (SCNS) and its short form (SCNS-SF34), the Cancer Rehabilitation Evaluation System (CARES), the Supportive Needs Screening Tool (SNST) (Bonevski et al., 2000; Boyes et al., 2009; Pigott et al., 2009; Johnsen et al., 2011). However, the assessment of the psychological needs of cancer patients in China started slightly later than in other countries; research in this field has mostly use translated and revised questionnaires developed in other countries. In particular, the SCNS-SF34 reportedly has good internal validity and reliability (Richardson et al., 2007; Carlson et al., 2012) and perform well in the Chinese population (Li et al., 2013; Choi et al., 2020). Whether it is the best fit for the Chinese population, however, remains uncertain. Localized and widely recognized reliable questionnaires have not been developed.

Thus, the aim of the present study was to construct the Psychological Needs of Cancer Patients Scale (PNCPS) with the Chinese population.

## Item Generation

As mentioned earlier, need refers to a lack and imbalance in the human tissue system. According to different standards, needs can be divided into different categories. Among them, Maslow’s hierarchy of needs theory (Maslow, 1943) is a representative theory of needs, and it has many implications for not only education and teaching but also the present study. Maslow’s hierarchy of needs divides human needs into five categories from low to high (like a ladder), as follows (Maslow, 1943; Koltko-Rivera, 2006):

**Physiological (survival) needs**: Seeks to obtain the basic necessities of life, such as food, water, air, sleep, and sex. They are the most important and powerful of human needs.

**Safety needs:** Seeks security through order and law.

**Belonging and love needs:** Seeks affiliation with a group.

**Esteem needs:** Seeks esteem through recognition or achievement.

**Self-actualization**: Seeks fulfillment of personal potential.

Generally speaking, when the needs of a certain level are relatively satisfied, they will develop to a higher level, and the pursuit of a higher level of needs will become the driving force for behavior. Correspondingly, the need to obtain basic satisfaction of needs is no longer an incentive.

Based on Maslow’s (1943) theory, open-ended questionnaires were conducted through semi-structured interviews completed by a representative sample of malignant tumor patients (see Table 1) to understand their psychological needs in depth and detail and to identify as many pertinent issues as possible from their perspective. In addition, four experts were invited for evaluation and consultation. This initial work was used to determine the dimensions and items of the preliminary questionnaire.

**TABLE 1:** Characteristics of participants: open-ended questionnaire.

Variable		*n*/(*M* ± *SD*)
Gender	Male	12
	Female	12
Marital status	Married	23
	Widowed	1
Tumor stage	I	6
	II	6
	III	6
	IV	6
Department	Radiotherapy general ward	2
	Gynecological oncology	9
	Urinary oncology	8
	Thoracic oncology	5
Age		56.46 ± 11.28

### Open-Ended Questionnaire

An open-ended questionnaire on the psychological needs of cancer patients was designed. The open-ended questionnaire mainly consists of five questions, which were corresponding with Maslow’s five needs. For example, the question about physiological needs was “How is your sleep recently? And what other physiological needs do you think are important to you?”

To ensure the data quality of the on-site interviews, interviewers were postgraduates selected from psychology majors and with experience in conducting on-site interviews. Before the formal interview, the interviewers were trained in the interview process and questionnaire content. Discussions and exchanges on the coding consistency of various topics were conducted to ensure the consistency of attitudes and interview content among interviewers.

A total of 24 patients (see Table 1) representing both genders and a range of ages, stages, and types of cancer were selected for in-depth face-to-face interviews. Written informed consent from the patients or their guardians was obtained before the interview. Centering on the topic, the interview was carried out with questions started with “how,” “what,” “when,” and “why.” Patients were asked to describe in detail the problems they had met in each stage of the disease, and specifically, the content and degree of the psychological needs, under what circumstances did the needs arise, and how satisfied these psychological needs were. The duration of the interview depends on the patients’ physical conditions and cooperation degree, and the duration of the interview for all patients ranges from 5 to 43 min. After the consent of the patients was obtained, the interviews were recorded and later coded.

In order to collect items as widely as possible, we extracted all the key words concerning “need” (whether satisfied or not) that appeared in the recordings and encoded them as sentences “I hope ….” Finally, an item pool containing 107 items was formed.

### Expert Consultation

The opinions of clinicians and experts (including an associate professor of psychology and three postgraduates in psychology) were obtained to develop the preliminary questionnaire. After their review and discussion on all 107 items, (1) items with similar meaning or overlapping content should be deleted or merged; (2) items with abstract and vague meanings should be modified to facilitate thinking by patients; and (3) optimize the language of all projects for easy reading and clear understanding, and items were managed.

### Preliminary Questionnaire Preparation

A total of 58 items were formed. After the initial questionnaire items were determined, a random number string was generated to reorder all items. All items started with “I hope …” and multiple-choice responses were anchored on a five-point scale (1 = completely inconsistent, 2 = less inconsistent, 3 = uncertain, 4 = more consistent, and 5 = completely consistent).

## Materials and Methods

Through open-ended questionnaire and expert consultation, we obtained the initial scale. Next, we obtained the patient’s performance on the scale through questionnaire method. A series of analysis, including item screening, exploratory factor analysis (EFA), and confirmatory factor analysis (CFA), were performed to develop the PNCPS.

### Participants and Sampling

The cluster sampling method was adopted, and Chongqing Cancer Hospital was selected as the survey site. Participants were randomly selected according to the following requirements: aged = 18 years; pathological diagnosis of cancer; intact cognitive function and normal ability to express understanding; adequate physical condition to complete the questionnaire; and able to provide signed informed consent and participate voluntarily. A total of 698 participants completed the questionnaire, of whom 599 (323 females) met the data screening criteria specifying that the continuous selection of the same option did not exceed 28 questions (Curran, 2016).

To construct the scale, 400 patients (Fabrigar et al., 1999; Lloret et al., 2014) were randomly selected from all subjects as Group 1, including 213 females, aged between 18 and 84 years (*M* = 51.87; *SD* = 12.53). The remaining 199 patients constituted Group 2, including 110 females, aged between 18 and 87 years (*M* = 53.73; *SD* = 12.85), were used to confirm the structure.

Additionally, the whole sample, including 599 patients (323 females), aged between 18 and 87 years (*M* = 52.49; *SD* = 12.66), was used to acquire information about psychometric properties. But only 441 patients’ scores of depression and anxiety were recorded in the hospital information system and were used for verification.

### Measures

#### Psychological Needs of Cancer Patients Scale

The preliminary version of the scale included 58 items. Responses were made on a five-point scale (1 = completely inconsistent, 2 = less inconsistent, 3 = uncertain, 4 = more consistent, and 5 = completely consistent). The higher the score, the more pressing the patient’s corresponding needs. As can be seen in the “Results and Discussion” section, the final version consisted of 23 items. Internal consistency (Cronbach’s alpha) was 0.83 and 0.85 for the confirmatory and the validation samples, respectively.

#### Depression Self-Rating Scale

Severity of depression was assessed using this 20-item scale (Zung, 1965) with responses made, as follows: 1 = no or very little time; 2 = a small amount of time; 3 = a lot of time; and 4 = most or all of the time. Ten items were reverse-scored. We added the scores of the 20 items to get the total raw score and then multiplied this by 1.25 to obtain the standard score. According to Chinese norms, the cutoff value of the Depression Self-rating Scale (SDS) standard score is 53 points, and 53–62 points indicate mild depression, 63–72 points indicate moderate depression, and 73 points or more indicate severe depression. The Cronbach α in this study was 0.91.

#### Anxiety Self-Assessment Scale

Severity of anxiety was measured using this 20-item scale (Zung, 1971) with responses made as follows: 1 = no or very little time; 2 = a small amount of time; 3 = a lot of time; and 4 = most or all of the time. Five items were reverse-scored. We added the scores of the 20 items to get the total raw score and then multiplied this by 1.25 to obtain the standard score. According to Chinese norms, the cutoff value of the Anxiety Self-Assessment Scale (SAS) standard score is 50 points, and 50–59 points indicate mild depression, 60–69 points indicate moderate depression, and 69 points or more indicate severe depression. The Cronbach α in this study was 0.89.

### Procedure

After patients were informed of the purpose of the study, written or verbal informed consent was obtained. Only participants who met the eligibility criteria were tested. The questionnaire was presented to each participant on a computer and during testing, and the testing personnel avoided the influence of irrelevant factors. All data were obtained between December 3, 2019, and April 5, 2020. The study was reviewed and approved by the Ethics Committee of Chongqing Cancer Hospital.

## Statistics

Firstly, Group 1 was used to explore the scale structure, which consisted of two steps: (1) screening for more efficient items using based on classical test theory (CTT); and (2) EFA was conducted. Secondly, CFA was conducted with Group 2 to confirm the rationality of the structure explored in the previous step. Finally, sample 3 was used to analyze psychometric properties of the scale.

The EFA and CFA were conducted with M Plus 8.6, and other analyses were processed with SPSS 22.0.

## Results and Discussion

### Item Screening

#### Internal Consistency Coefficient

We calculated the Cronbach α coefficient of the scale. If the value of the new Cronbach α coefficient do not decrease after deleting an item, then the relationship between this question and other questions in the scale is weak, and this item should be considered for exclusion. In this step, 12 items was eliminated: 11, 14, 16, 19, 24, 26, 30, 40, 50, 55, 56, and 57. After seven items were deleted, the Cronbach α of the 46 items was 0.88.

#### Corrected Item–Total Correlation

We considered items with low corrected item–total correlations as problematic. And seven items (3, 10, 18, 46, 47, 48, and 53) were excluded from subsequent analyses.

In addition, the averages of 39 items range between 3.14 and 4.76, and the skewness range between −2.24 and −0.13.

### Exploratory Factor Analysis

Based on the results of Item Screening, 39 items were included in the EFA. Considering the non-normal distribution of item scores, EFA was run using the robust weighted least square mean and variance (WLSMV) estimation (Beauducel and Herzberg, 2006; Muthén and Muthén, 2013). Item responses were treated as categorical variables, and oblique rotations using the GEOMIN method were generated.

During the EFA process, items that load strongly (>0.35) onto factors were retained. Also, a model was accepted with at least three items for each dimension.

The results of EFA show that the eight-factor model fits well: χ^2^ = 784.33, *df* = 457, comparative fit index (*CFI*) = 0.94, Tucker–Lewis index (*TLI*) = 0.91, root mean square error of approximation (*RMSEA*) = 0.04, and standardized root mean square residual (*SRMR*) = 0.05; but one of the factors only contains two items. Based on the eight-factor model, nine items (2, 4, 5, 7, 9, 20, 21, 22, and 25) were removed due to factor loads below 0.35. The remaining 30 items were transferred to the next EFA.

Using the same process, we repeated the factor analysis two more times to explore the best fitting latent structure. In the first round, three items (6, 8, and 34) were removed because the coefficient was below 0.35. In the second round, four items (1, 23, 36, and 38) were removed as the coefficient was below 0.35.

Ultimately, the six-factor model consisting of 23 items fits well: χ^2^ = 290.38, *df* = 130, *CFI* = 0.96, *TLI* = 0.91, *RMSEA* = 0.06, and *SRMR* = 0.05, and each factor contains at least three items. Combined with the meaning of each item, the six factors were defined as follows: value and esteem, independence and control, mental care, disease care, belonging and companionship, and security. And the corresponding eigenvalues for sample correlation matrix were 5.61, 1.98, 1.81, 1.74, 1.43, and 1.28, respectively. Factor loads are shown in Table 2.

**TABLE 2:** EFA: 23 items and corresponding factor loads.

Item	F1	F2	F3	F4	F5	F6	Content
15	**0.70**						I hope to do some outdoor activities like walking and playing ball
13	**0.66**	−0.14					I hope to know the diagnosis of my disease
12	**0.58**						I hope to reconsider the meaning and purpose of life
17	**0.54**						I hope there are fewer people who know about my health condition
43	**0.40**		0.19	0.32			I hope I can take part in social work and continue to make use of my value
29	0.17	**0.69**					I hope to stay away from death, from things that remind me of death
27	0.25	**0.65**					I hope to understand death, face death, and think about death
31		**0.63**					I hope it is convenient to walk out
33		**0.63**					I hope to have more private space
32		**0.47**	0.19			0.14	I hope to get help financially
28		**0.43**					I hope I can make my own decisions on whether to receive treatment and what treatment to receive
37			**0.79**				I hope I can vent sadness, fear, anger, or other negative emotions
39			**0.66**	0.30			I hope to know about changes in my condition and related information as soon as possible
35	−0.15	0.35	**0.53**				I hope I can feel useful to my family
44				**0.74**			I hope the pain of eating can be eased
42				**0.72**			I hope to acquire some knowledge about my disease
41	0.28	0.16	0.23	**0.39**			I hope to talk to a professional psychologist about my mental state
49					**1.06**		I hope I can live a fulfilling life
51					**0.49**		I hope I can spend more time at home
45				0.27	**0.42**	−0.16	I hope my family can stay with me often
52						**0.79**	I hope to be able to complete daily activities independently
54	0.22					**0.75**	I hope I can confide in others
58		0.20				**0.42**	I hope the living conditions could be better

*EFA, exploratory factor analysis.*

*Bold values emphasize the loads of items on the corresponding factor, to distinguish from other cross-loads.*

It is important to note that the factor load of item 49 onto Factor 5 is 1.06. Due to the specificity of the group of cancer patients, the distribution of the score is biased, which led to the result.

It should also be noted that there were still some obvious cross-loading associated with a few items. Item 43 not only loads on value and esteem but also loads high on disease care. Item 39 loads on mental care and on disease care.

### Confirmatory Factor Analysis

**FIGURE 1:**
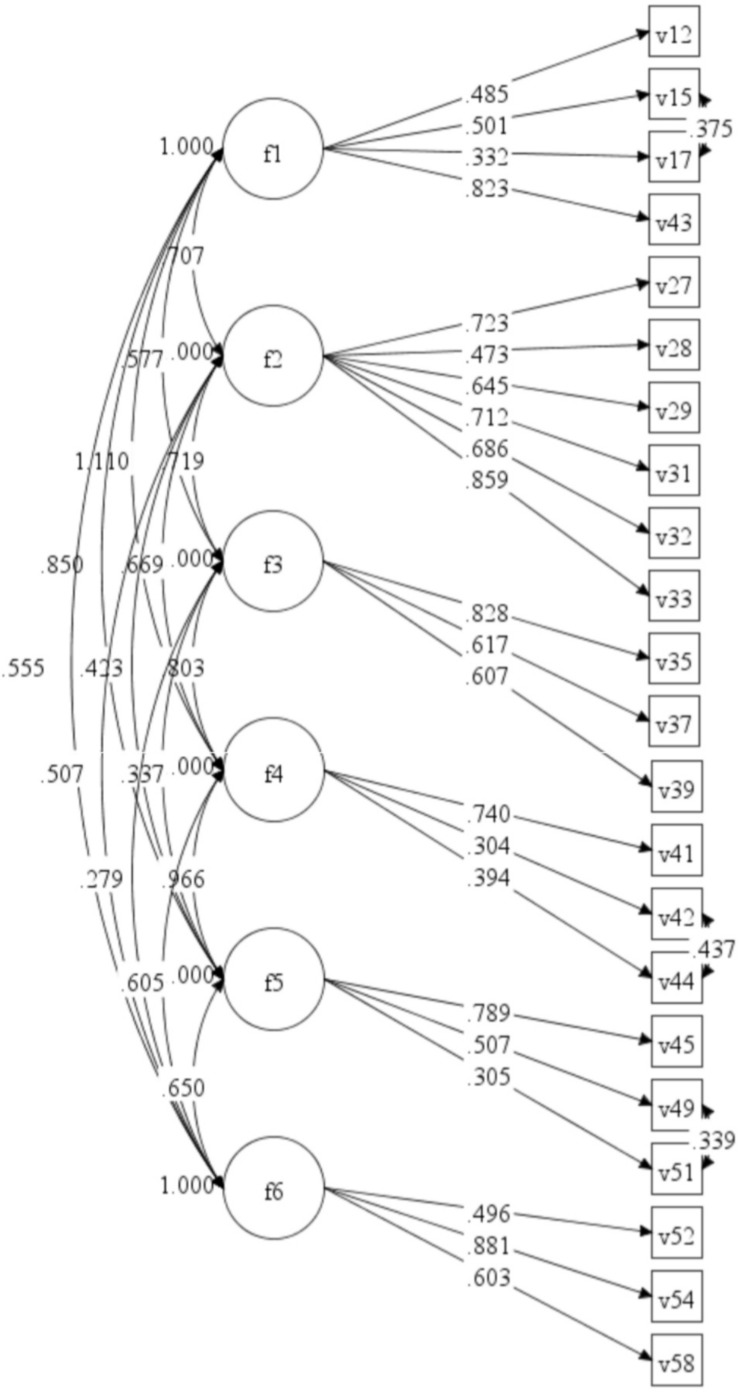
The six-factor model and its standardized factor loading.

To confirm the six-factor structural model constructed in Group 1, CFA was performed using the WLSMV estimation. For this six-factor model in Group 2, χ^2^ = 459.17, *df* = 194, *CFI* = 0.88, *TLI* = 0.86, and *RMSEA* = 0.08. We modified the model by adding three correlations: items 15 and 17, items 42 and 44, and items 49 and 51. The results showed that the six-factor model constructed in Group 1 was consistent with the data in Group 2, χ^2^ = 363.99, *df* = 191, *CFI* = 0.92, *TLI* = 0.90, and *RMSEA* = 0.07. The normalized coefficients for each path and factor loads in Group 2 are shown in Figure 1.

The four-item factor loads were below 0.40, which is essentially related to the Cronbach α coefficient.

### Psychometric Properties of Psychological Needs of Cancer Patients Scale

#### Internal Consistency

Inter-correlations between subscales ranged from 0.20 to 0.53, all statistically significant at the 0.01 level (see Table 3).

**TABLE 3:** Correlations of the factors.

	F1	F2	F3	F4	F5	F6	PNCPS
F1	1						
F2	0.53**	1					
F3	0.28**	0.43**	1				
F4	0.40**	0.46**	0.31**	1			
F5	0.32**	0.32**	0.26**	0.28**	1		
F6	0.33**	0.38**	0.22**	0.32**	0.20**	1	
PNCPS	0.76**	0.85**	0.59**	0.66**	0.53**	0.58**	1
Age	0.06	0.03	0.02	−0.01	0.01	0.04	0.04
SAS	−0.10*	−0.10*	−0.07	−0.04	0.01	−0.07	−0.10*
SDS	0.02	−0.02	0.00	−0.04	0.00	−0.07	−0.02

PNCPS, Psychological Needs of Cancer Patients Scale; SAS, Anxiety Self-Assessment Scale; SDS, Depression Self-Rating Scale. **Correlation is significant at the 0.01 level (two-tailed). *Correlation is significant at the 0.05 level (two-tailed).

In terms of internal consistency and reliability, the Cronbach α of the six subscales of value and esteem, independence and control, mental care, disease care, belonging and companionship, security, and the entire scale were 0.62 (five items), 0.71 (six items), 0.61 (three items), 0.42 (three items), 0.55 (three items), 0.49 (three items), and 0.84, respectively.

When the number of items is too small, it will violate the assumption of tau-equivalence, which underlies the Cronbach α, and will underestimate reliability (Kottner and Streiner, 2010; Tavakol and Dennick, 2011; Taber, 2017). In a sense, combined with the low-factor-loading, they indicate the multiple aspects of need and the relative independence of each factor. Given that the Cronbach α of the entire scale was 0.84, we consider it as a high degree of internal consistency.

#### Correlation With Anxiety Self-Assessment Scale and Depression Self-Rating Scale

Pearson correlations were conducted to explore the associations between PNCPS and the standard measures of depression and anxiety. The results showed that there was no significant correlation, but SAS was significantly correlated with value and esteem, independence and control, and PNCPS, and correlations were weak (*r*s = 0.10) (see Table 3).

#### Influence of Gender and Age on Psychological Needs of Cancer Patients Scale

An independent samples *t*-test showed that there were significant differences in psychological need scores between males and females. Specifically, males scored higher than females in value and esteem [*t*(597) = 3.14, *p* = 0.00 (two-tailed test)], independence and control [*t*(596) = 2.32, *p* = 0.02 (two-tailed test)], and PNCPS [*t*(597) = 2.54, *p* = 0.01 (two-tailed test) see Table 4].

**TABLE 4:** Influence of gender on PNCPS.

	Gender	*M*	*SD*	*t*	*df*	*p*
F1	Male	21.53	2.75	3.14**	597	0.002
	Female	20.79	3.02			
F2	Male	25.32	3.58	2.32*	596	0.021
	Female	24.60	4.02			
F3	Male	13.04	1.84	0.77	597	0.44
	Female	12.92	1.95			
F4	Male	12.88	1.88	0.64	597	0.525
	Female	12.78	1.94			
F5	Male	13.24	1.65	0.32	597	0.751
	Female	13.20	1.54			
F6	Male	13.18	1.73	1.96	597	0.051
	Female	12.88	2.04			
PNCPS	Male	99.19	9.53	2.54*	597	0.011
	Female	97.16	9.91			

PNCPS, Psychological Needs of Cancer Patients Scale. **Correlation is significant at the 0.01 level (two-tailed). *Correlation is significant at the 0.05 level (two-tailed).

There was also no correlation between age and psychological need scores (see Table 3), which indicates the invariance across age of the scale.

## General Discussion

We conducted this study to develop a reliable and effective scale to measure the psychological needs of patients with malignant tumors. Based on Maslow’s (1943) hierarchy of needs theory, we conducted semi-structured interviews with a sample of representative malignant tumor patients to understand their psychological needs in depth and detail, and in doing so, we identified a large range of concerns. Experts in relevant fields also participated in evaluation and consultation. Based on this work, the dimensions and items of the preliminary questionnaire were preliminarily determined, and a final scale consisting of 23 items and six dimensions was constructed running with EFA. The results of the next CFA showed that the six-factor structure fitted the data well. In terms of reliability and validity, the Cronbach α showed that the scale as a whole has high internal validity. Additionally, the scale also showed invariance across age. Therefore, despite some minor flaws, we conclude that the current 23-item scale is a reliable, theory-based tool for measuring the psychological needs of patients with malignant tumors.

Four factors extracted from our research results (value and esteem, independence and control, belonging and companionship, and security) highlight the middle three needs of Maslow’s hierarchy of needs, namely, the needs for safety, esteem, and belonging and love. There is only one item concerning basic physiological need, “I hope the pain of eating can be eased.” This is in line with the situation of cancer patients. Faced with the direct threat of cancer, patients can do only very limited things, and other lower-level needs are more likely to be met.

Our research also identified two additional factors relating to the needs of cancer patients, namely, mental care and disease care. Similarly, in Zebrack’s (2009) study, nearly all cancer patients (98.1%) expressed a need for information about the disease, treatment, and prognosis. A study of myeloma patients by Yogaparan et al. (2009) showed that more than two-thirds of older patients (aged over 60 years) needed to know about the life-prolonging effects of treatment (77%) and the side effects of treatment (67%), but more than 10% of patients thought they were given too little information about these two issues. According to Sollner et al. (2004), 31% of patients showed moderate-to-severe anxiety and depression in the early stages of treatment, and 41% strongly expressed the need for psychological support. This is both understandable and consistent with Maslow’s hierarchy of needs theory.

In addition, there is the very interesting aspect of death. Our results show that cancer patients need to know about and face death. But in the context of Chinese culture, people’s views on death have been influenced by Confucianism, Taoism, Buddhism, and other perspectives for a long time. In China, people always take a negative and veiled attitude toward death and do not mention death in words, considering it as a symbol of misfortune and fear (Ding, 2000). However, death is a problem that cancer patients, especially those at an advanced stage, have to face. This contradiction explains the necessity of hospice care and death education. Therefore, we suggest that we should pay attention to the care of the dying patients and implement death education, so that they can live without pain and die with dignity.

Despite a number of studies from the perspective of self-determination theory (Ryan and Deci, 2000), the satisfaction of basic psychological needs seems to predict a decrease in anxiety and depression (Wei et al., 2005; Yu et al., 2016). However, the same result was not found in this research. There was no significant correlation between psychological needs and depression and only a slight negative correlation between psychological needs and anxiety in patients with malignant tumors. One possible explanation is that different definitions of psychological needs and the tools used to measure them, as well as different groups of people, will lead to differing results. In addition, depression and anxiety related to cancer are different from general depression and anxiety. The occurrence of depression and anxiety is a complex process and not caused by one single factor. In addition to psychological factors, the development of depression and anxiety is also related to therapeutic drugs, hormones, and other chemicals (Wu and Xiao, 2011). Finally, the difference may also be caused by the unsatisfactory testing environment. Due to economic and time constraints, the questionnaire was not completed by patients individually under the supervision of professionals, so it was difficult to ensure that all patients responded carefully.

The development of a scale to assess the psychological needs of malignant tumor patients in China is of great significance. Only by understanding the content of patients’ most urgent needs for help can we provide targeted help to cancer patients, make the optimal allocation of medical service resources, carry out mental health education and intervention, prevent possible psychological problems, improve quality of life, and achieve the simultaneous treatment of body and mind. It is of great practical significance to promote mental health and prolong the survival of patients with malignant tumors.

There are regional differences in the incidence levels of malignant tumors, and the psychological needs of patients with malignant tumors in different regions may also be different. Research in China in this field has not developed a localized, widely recognized, or reliable questionnaire. Participants in this study were from the largest cancer hospital in southwest China and may represent the whole of China to some extent. The PNCPS may become a widely used questionnaire as it takes the Chinese cultural background into account and fills existing gaps in this field. In this way, the PNCPS may contribute to a more accurate understanding of the psychological needs of Chinese, even the whole word’s, malignant tumor patients and provide a basis for the formulation of targeted and effective measures.

This study also has some limitations. First, the results are based on data from patients in Chongqing Cancer Hospital, China, and the same results may not be obtained in other countries. Second, this was a cross-sectional survey, and the retest reliability of the scale has not been examined. Thus, the time stability of the scale remains to be confirmed. Third, as terminal cancer patients suffer great pain, it is difficult for them to support and cooperate with research, and terminal cancer patients were less represented in the sample, which may have some influence on the structure of the questionnaire. In conclusion, the validity of the PNCPS needs to be tested in more studies, and the scale needs to be revised and improved accordingly.

## Data Availability Statement

The original contributions presented in the study are included in the article/Supplementary Material, further inquiries can be directed to the corresponding author/s.

## Ethics Statement

The studies involving human participants were reviewed and approved by the Ethics Committee of Chongqing Cancer Hospital. Written informed consent for participation was not required for this study in accordance with the national legislation and the institutional requirements.

## Author Contributions

Y-LT, LX, and JL led the design and implement of the study, including the literature search, analysis, and interpretation of the data. YC and FL led the drafting, writing, and revising of manuscript. All authors contributed to the questionnaire construction, elaboration, data collection, analysis, interpretation of data, and read and approved the final version of the work to be published and agreed to be accountable for all aspects of the work in ensuring that any question to the accuracy of the work is appropriately investigated and resolved.

## Conflict of Interest

The authors declare that the research was conducted in the absence of any commercial or financial relationships that could be construed as a potential conflict of interest.
