# Supplementary material for: The Development and Validation of the Psychological Needs of Cancer Patients Scale
Source: Front Psychol. 2021 Jun 3;12:658989. doi: 10.3389/fpsyg.2021.658989 (PMC8209331; doi:10.3389/fpsyg.2021.658989)
Supplement: Supplementary file 1 [file Data_Sheet_1.ZIP › supplementary materials/1.outline of open-ended questionnaire.docx]

**半结构式访谈提纲**

开场：自我介绍，说明知情同意书。

请谈一下患病以来，您的身体、心理和生活发生了哪些变化？您对此有什么感受？或者从任何您想说的地方开始都可以。

1. 您最近的睡眠怎么样（具体：睡眠时间、质量、精力等）？（如较差：询问原因、应对方法、希望获得的帮助）

睡眠是保证正常生活的一个必要条件，是一种最基本的需要。对于这一类需要您认为还有哪些是对您比较重要的、希望能够得到重视和关注的？（如饮食、温度、声音）

1. 您的治疗费用是否会对您产生经济负担？（如有经济负担：询问原因、应对方法、希望获得的帮助）

对于这一类需要您认为还有哪些是对您比较重要的、希望能够得到重视和关注的？（如工作、保险、政府社会、医院方面）

1. 您在治疗期间是否感受到来自家人在情感上的支持？ （如有：在什么样的情况下如何满足。如没有：询问原因、应对方法、希望获得的帮助）

对于这一类需要您认为还有哪些是对您比较重要的、希望能够得到重视和关注的？（如同事朋友、病友组织、医护人员）

1. 您觉得自己在身体、生活等方面做出自主决定的能力怎么样？（如有：在什么样的情况下如何满足。如没有：询问原因、应对方法、希望获得的帮助）

对于这一类需要您认为还有哪些是对您比较重要的、希望能够得到重视和关注的？（如在工作、生活中取得成就，获得他人认可与尊重）

1. 您对于自己想做的事情有什么想法吗?在您目前看来，你对自己个人有什么看法呢？（自我实现的需要，是指人高层次的发展需要，即成为自己希望成为的那个人的需要，如对生命意义、个人价值的追求。）

对于这一类需要您认为还有哪些是对您比较重要的、希望能够得到重视和关注的？（如理想、人生目标、自我价值、发挥潜能）

您还有其他没有提到需要补充的内容吗？

十分感谢您的配合，祝您生活愉快！
