# Supplementary material for: The Development and Validation of the Psychological Needs of Cancer Patients Scale
Source: Front Psychol. 2021 Jun 3;12:658989. doi: 10.3389/fpsyg.2021.658989 (PMC8209331; doi:10.3389/fpsyg.2021.658989)
Supplement: Supplementary file 1 [file Data_Sheet_1.ZIP › supplementary materials/2.item pool.pdf]

1. 需要更丰富的食物
2. 需要可以继续吸烟
3. 需要可以继续饮酒
4. 需要减少进食的痛苦
5. 需要额外营养保健品支撑身体
6. 需要更好的睡眠
7. 需要更好的居住环境
8. 需要使用药物来减少/减轻不适感
9. 需要尽可能延长生命
10. 需要病情得到控制、不再恶化
11. 需要有人协助进食、穿衣、洗漱等
12. 需要远离死亡
13. 需要减少疼痛
14. 需要性生活

15. 需要更多的时间在家里度过
16. 需要独立完成日常活动的能力
17. 需要周围社区宣传医疗保健知识
18. 需要周围社区配备完备的健身设施
19. 需要可以及时得到病人的病情变化及有关信息
20. 需要在检查前了解该检查的作用及注意事项
21. 需要不去担心自己会丧失或部分丧失活动能力
22. 需要医生告知肿瘤已经得到控制或正在康复
23. 需要对自己疾病的发展有所心理准备
24. 需要自己做出是否接受治疗及何种治疗方式的决定
25. 需要了解疾病对性生活的影响
26. 需要尽可能的延长生命
27. 需要知道疾病诊断的结果
28. 需要了解疾病相关知识
29. 需要在外面进行散步、打球等活动
30. 需要更好的住房条件
31. 需要医保报销更多治疗费用
32. 需要出行方便

33. 需要亲人朋友对自己的经济援助
34. 需要有人关心
35. 需要了解当地有关的癌症互助组织
36. 需要亲戚朋友同事能经常来探望自己
37. 需要与有相同经历的病友相互交流心得体会
38. 需要家人的精神支持和关爱
39. 需要参加团体活动

40. 需要更好的邻里关系
41. 需要被接纳
42. 需要与家人交流
43. 需要家人关爱
44. 需要感到对家庭有用
45. 需要感到自己不被抛弃
46. 需要子女经常看望
47. 需要经常跟亲戚朋友来往
48. 需要家人陪床
49. 需要亲戚、朋友常常看望自己
50. 需要周围人的鼓励与支持
51. 需要和人聊天
52. 需要亲友、同事的鼓励
53. 需要周围人不以异样的眼光看待自己
54. 需要参与心理互助小组
55. 需要跟子女关系好
56. 需要家庭和谐、减少矛盾
57. 需要与外界交流
58. 需要得到别人理解
59. 需要在有困难时能得到帮助

60. 需要别人信任
61. 需要得到别人尊重
62. 需要避免被同情、怜悯
63. 需要家庭有事时和自己商量
64. 需要子女采取自己意见
65. 需要外形和原来一样
66. 需要尽量少的人知道自己的病情
67. 需要尽量减少给亲友带来的影响
68. 需要更多的私密空间
69. 需要时间适应疾病带给自己身体样貌上的改变
70. 需要继续工作以增加收入
71. 需要减少家庭负担
72. 需要身体能够执行日常功能
73. 需要参与社会工作以继续发挥余热
74. 需要重新思考生活的意义和目的
75. 需要找到癌症背后的正面意义
76. 需要继续想做而没有做成的事情
77. 需要继续从事之前的工作以获得乐趣
78. 需要身体允许自己坚持兴趣爱好
79. 需要做自己感兴趣的事情
80. 需要做一些对他人有利的事情
81. 需要从事创造性的工作
82. 需要生活充实

83. 需要医务人员对自己的不适作出反应并采取措施
84. 需要医生消除自己对治疗、药物等方面的疑虑

85. 需要能够随时联系医生
86. 需要医护人员不出现失误
87. 需要医生在出院前能告知复诊的具体时间和详细内容
88. 需要医生指导做什么能使疾病好转
89. 需要医生对家属进行如何护理的具体技术和知识指导
90. 需要医生尽可能减轻癌痛
91. 需要在住院期间各种医学检查都能快速、准确、不出差错
92. 需要护士能不出差错，技术精湛，熟练
93. 需要护士经常查看病房情况，及时向医生反映病情
94. 需要护士陪伴
95. 需要护士抚触
96. 需要医生抚触
97. 需要与护士交流
98. 需要和心理医生交谈
99. 需要得到护士更多的注意
100. 需要护士能够给予最好的照顾
101. 需要能够在与护士的交流中表达一些负面情绪
102. 需要医生尽快告知有关疾病的化验检查结果和意义

103. 需要专业人员进行团体心理辅导、开设心理知识讲座
104. 需要参与组织团体活动（如拓展训练、心理沙龙、心理知识竞赛等）
105. 需要进行心理咨询
106. 需要与心理工作者讨论自己的心理问题
107. 需要有专业机构或心理工作者提供心理服务
