# Supplementary material for: The Development and Validation of the Psychological Needs of Cancer Patients Scale
Source: Front Psychol. 2021 Jun 3;12:658989. doi: 10.3389/fpsyg.2021.658989 (PMC8209331; doi:10.3389/fpsyg.2021.658989)
Supplement: Supplementary file 1 [file Data_Sheet_1.ZIP › supplementary materials/3.58-item-scale.docx]

**恶性肿瘤患者心理需求问卷**

您好，我们是重庆市肿瘤医院的科研人员，想通过这份调查问卷，了解您的需要，作为以后提供住院患者帮助的重要资料。

请根据您过去患病以来的经验与事实，阅读一下提问回答问题。请从题目右边的5个选项：1~5（1=完全不符合，2=比较不符合，3=不确定，4=比较符合，5=完全符合）的5个空格中，勾选出您觉得最符合您情况的答案，答案无好坏、对错之分，只要符合您的情况即可。请不要遗漏任何一题。

对您的回答我们仅作为科研之用，也会对您的回答进行严格保密，请不要有任何顾虑。谢谢您的合作！

课题组：熊琳、林芳岩

联系方式：[739700897@qq.com](mailto:739700897@qq.com)（邮箱）

023-65079364（电话）

| **1=完全不符合，2=比较不符合，**  **3=不确定，4=比较符合，5=完全符合** | | **1** | **2** | **3** | **4** | **5** |
| --- | --- | --- | --- | --- | --- | --- |
| 1. | 我希望在我感觉不舒服的时候，医务人员能及时做出反应，做出有效的应对措施 |  |  |  |  |  |
| 2. | 我希望不要被同情、可怜 |  |  |  |  |  |
| 3. | 我希望身体不舒服的感觉（头晕目眩、干呕等）可以减少/减轻一些 |  |  |  |  |  |
| 4. | 我希望亲人朋友能常来探望自己 |  |  |  |  |  |
| 5. | 我希望有专业的人来进行团体心理辅导、开设心理知识的讲座等 |  |  |  |  |  |
| 6. | 我希望家里有什么事时能和我商量、听听我的意见 |  |  |  |  |  |
| 7. | 我希望能和左邻右舍相处得更好 |  |  |  |  |  |
| 8. | 我希望能做自己感兴趣的事情 |  |  |  |  |  |
| 9. | 我希望能尽量减少给亲人朋友带来的负面影响 |  |  |  |  |  |
| 10. | 我希望身体上的疼痛（癌痛）能减轻一些 |  |  |  |  |  |
| **1=完全不符合，2=比较不符合，**  **3=不确定，4=比较符合，5=完全符合** | | **1** | **2** | **3** | **4** | **5** |
| 11. | 我希望有符合需求的性生活 |  |  |  |  |  |
| 12. | 我希望重新考虑生活的意义和目的 |  |  |  |  |  |
| 13. | 我希望可以知道我的疾病诊断结果 |  |  |  |  |  |
| 14. | 我希望医院能指导做些什么会让我的病情/症状好转一些（护理的具体技术、知识指导等） |  |  |  |  |  |
| 15. | 我希望能在室外做一些散步、打球等活动 |  |  |  |  |  |
| 16. | 我希望可以不用担心自己会丧失或部分丧失活动能力 |  |  |  |  |  |
| 17. | 我希望知道我病情的人可以尽量少一些 |  |  |  |  |  |
| 18. | 我希望周围的人不要用异样的眼光来看我 |  |  |  |  |  |
| 19. | 我希望在我有困难的时候能得到帮助 |  |  |  |  |  |
| 20. | 我希望有和其他人（病友、亲人等）交流的机会 |  |  |  |  |  |
| **1=完全不符合，2=比较不符合，**  **3=不确定，4=比较符合，5=完全符合** | | **1** | **2** | **3** | **4** | **5** |
| 21. | 我希望可以适应疾病给我带来的身体、样貌上的改变 |  |  |  |  |  |
| 22. | 我希望家里人都和睦相处，少一些争吵（和孩子关系好） |  |  |  |  |  |
| 23. | 我希望可以继续抽烟或喝酒 |  |  |  |  |  |
| 24. | 我希望了解疾病对身体器官功能有什么影响 |  |  |  |  |  |
| 25. | 我希望睡眠可以更好 |  |  |  |  |  |
| 26. | 我希望被周围的人接纳、理解和信任 |  |  |  |  |  |
| 27. | 我希望可以了解死亡、正视死亡与思考死亡 |  |  |  |  |  |
| 28. | 我希望可以自己做出是否接受治疗、接受什么治疗的决定 |  |  |  |  |  |
| 29. | 我希望可以远离死亡，避免让我想到死亡的事情和东西 |  |  |  |  |  |
| 30. | 我希望医务人员可以技术熟练、不要出现失误 |  |  |  |  |  |
| **1=完全不符合，2=比较不符合，**  **3=不确定，4=比较符合，5=完全符合** | | **1** | **2** | **3** | **4** | **5** |
| 31. | 我希望可以出行方便 |  |  |  |  |  |
| 32. | 我希望在经济上能得到帮助 |  |  |  |  |  |
| 33. | 我希望有更多自己的私密空间 |  |  |  |  |  |
| 34. | 我希望可以继续做之前没有完成的事情 |  |  |  |  |  |
| 35. | 我希望可以感到对家里有用 |  |  |  |  |  |
| 36. | 我希望减少对家里的负担 |  |  |  |  |  |
| 37. | 我希望可以发泄难过、害怕、生气或者其他的负面情绪 |  |  |  |  |  |
| 38. | 我希望可以在检查前了解这个检查的作用和注意事项 |  |  |  |  |  |
| 39. | 我希望能尽快知道我的病情变化和有关的信息 |  |  |  |  |  |
| 40. | 我希望得到医生的解释、保证，来消除我对治疗、药物等方面的疑虑 |  |  |  |  |  |
| **1=完全不符合，2=比较不符合，**  **3=不确定，4=比较符合，5=完全符合** | | **1** | **2** | **3** | **4** | **5** |
| 41. | 我希望可以和专业的心理工作者谈一谈我的心理状态 |  |  |  |  |  |
| 42. | 我希望可以了解一些和我的疾病有关的知识 |  |  |  |  |  |
| 43. | 我希望可以参加社会工作，继续发挥我的价值 |  |  |  |  |  |
| 44. | 我希望可以减少进食的痛苦 |  |  |  |  |  |
| 45. | 我希望家人可以常常陪我 |  |  |  |  |  |
| 46. | 我希望可以和医生谈一谈我的病情发展和变化 |  |  |  |  |  |
| 47. | 我希望得到周围人（包括亲人）的精神支持和言语鼓励 |  |  |  |  |  |
| 48. | 我希望不要被周围的人抛弃 |  |  |  |  |  |
| 49. | 我希望可以生活充实 |  |  |  |  |  |
| 50. | 我希望在住院期间得到各种医学检查快速、准确 |  |  |  |  |  |
| **1=完全不符合，2=比较不符合，**  **3=不确定，4=比较符合，5=完全符合** | | **1** | **2** | **3** | **4** | **5** |
| 51. | 我希望可以有更多的时间是在家里度过的 |  |  |  |  |  |
| 52. | 我希望可以独立完成日常活动 |  |  |  |  |  |
| 53. | 我希望饮食可以更丰富、味道更好 |  |  |  |  |  |
| 54. | 我希望可以向其他人倾诉 |  |  |  |  |  |
| 55. | 我希望知道更多的方法来得到医疗保健知识 |  |  |  |  |  |
| 56. | 我希望可以尽快知道有关疾病的化验检查结果和意义 |  |  |  |  |  |
| 57. | 我希望我的病情可以得到控制、不再恶化（尽可能延长生命） |  |  |  |  |  |
| 58. | 我希望居住条件可以更好一些 |  |  |  |  |  |

姓名： 性别： 科室： 床号：
