# Supplementary material for: The Development and Validation of the Psychological Needs of Cancer Patients Scale
Source: Front Psychol. 2021 Jun 3;12:658989. doi: 10.3389/fpsyg.2021.658989 (PMC8209331; doi:10.3389/fpsyg.2021.658989)
Supplement: Supplementary file 1 [file Data_Sheet_1.ZIP › supplementary materials/CWS_Editorial_Certificate.pdf]

EDITORIAL CERTIFICATE

This document certifies that the manuscript below was edited for correct English language usage, grammar, punctuation and spelling by qualified native English speaking editors at Charlesworth Author Services.

Paper Title:

The Development and Validation of the Psychological Needs of Cancer Patients Scale

Author:

芳岩 林

Date certificate issued:

January 20, 2021

cwauthors.com
