# Supplementary material for: The Development and Validation of the Psychological Needs of Cancer Patients Scale
Source: Front Psychol. 2021 Jun 3;12:658989. doi: 10.3389/fpsyg.2021.658989 (PMC8209331; doi:10.3389/fpsyg.2021.658989)
Supplement: Supplementary file 1 [file Data_Sheet_1.ZIP › supplementary materials/Ethics Committee Approval.pdf]

重庆市肿瘤医院伦理委员会审批件

2019 年伦审（177 ）号

审查内容	2019 年医学科研计划项目（目录见附件）

伦理委员会审批意见：

经伦理委员会审查，科外部所提交的 79 项医学科研计划项目的研究方案等资料符合医学伦理原则和赫尔辛基宣言的各项要求，伦理委员会成员以投票表决方式，决定同意其中 79 项医学科研计划项目在院内开展，分别为：

1 肿瘤化疗相关性恶心呕吐预测管理智能体系的构建与应用

2 三孔 NOSES 手术在腹腔镜结直肠癌根治术中的临床应用研究

3 基于 miR-138 的卵巢癌诊疗方法研究与应用

4 “端-端、侧-侧、嵌入吻合”方法对于食管癌术后返流影响的研究

5 超声造影定量参数分析预测前列腺癌侵袭性研究

6 术中腹腔热灌注化疗应用于可切除胰腺癌的临床研究

7 基于木犀草素的卵巢癌诊疗方法的研究与应用

8 恶性肿瘤临床大数据智能诊疗平台研发及应用

9 肿瘤仿制药 BE 受试者快速分子标记及其特征性信息数字指纹转换技术研发与应用

10 高通量靶向测序 panel 的研发以及在弥漫大 B 细胞性淋巴瘤分子分型的应用

11 金纳米笼——温敏脂质体靶向控释载药系统的制备及在抗乳腺癌中的研究

12 circ-MTCL1 在结直肠癌细胞转移中的作用及机制研究

13 沙丁胺醇联合 Rock 抑制剂对气道平滑肌的舒张作用的协同效应研究

14 IDH1 突变胶质瘤白质纤维束异化特征的实验研究

15 内嗅皮层--海马 CA1 区长距离抑制性投射参与老年术后认知功能障碍的发生机制

16 miR-221-3p 经下调 PARP1 抑制 NF-κB 通路改善三阴性乳腺癌恶性生物学行为的机制研究

地址：重庆市沙坪坝区汉渝路 181 号　　邮编：400030
电话：023-65327159　　传真:023-65305442　　Email:65305442@163.com

17 SRC-3 通过乙酰化修饰调控 JAK/STAT 信号通路在结直肠癌发生发展中的机制研究

18 抗增殖蛋白 Prohibitin 抑制细胞周期检测点基因 GADD45a 在膀胱癌表达的机制研究

19 snoRNA47 通过调控 PTEN 进而促进肺癌细胞增殖的分子机制研究

20 核仁 GTP 结合蛋白 GNL3 通过促进结肠癌干性获取从而调控结肠癌侵袭转移的分子机制研究

21 RGD 修饰的纳米粒递送紫杉醇靶向肿瘤微环境 TGF-β smad 通路，增强 PD1 及 PD-L1 治疗肺癌的作用机制研究

22 Al18F 标记的双氨基酸类小分子探针用于脑胶质瘤早期诊断的研究

23 索拉非尼对降低 HCC 合并 MVI 患者术后早期复发的前瞻性、随机对照临床研究

24 局部晚期宫颈癌腔内联合组织间插植近距离放疗的临床研究

25 CT 图像影像组学信息联合临床特征及放疗剂量学参数预测非小细胞肺癌放射性肺炎的发生

26 3D 打印个体化模具在宫颈癌近距离放疗中的应用研究

27 基于深度迁移学习的肺部 CT 鳞、腺癌分类及基因突变预测

28 EGFR-TKI 联合贝伐珠单抗治疗一线靶向治疗后缓慢进展肺癌的临床研究

29 去阿片化快通道麻醉促进胸腔镜肺癌患者早期康复和免疫功能优化的临床研究

30 中晚期宫颈癌同步放化疗患者症状评估量表构建及症状群纵向研究

31 PET-CT 联合 4D-CT 在胸段食管癌放疗中的应用

32 癌症患者 PICC 相关性血栓风险评估指标体系的研制及系统评价

33 肿瘤化疗相关性恶心呕吐中医分层护理方案的构建及实证研究

34 基于超声造影的超微血管架构重建成像用于早期前列腺癌诊断研究

35 基于信息化的癌症患者心理困扰分层管理模式的构建及叙事护理干预实证研究

36 基于 WARM 模型的早期姑息治疗技术在肺癌患者中的应用

37 EDGE 放射手术肿瘤治疗系统在非手术治疗肝癌患者中的应用价值研究

38 图式理论对肺癌术后化疗病人的感知控制与疾病不确定感的影响
39 恶性肿瘤合并静脉栓塞性疾病数据库的建立与临床应用
40 重庆市基层医院实验室生物安全现状及对策研究
41 药师参与癌痛联合门诊的药物治疗管理服务模式的构建及应用
42 儿童肿瘤放疗跌倒/坠床管理体系的构建
43 两种风险评估模型对恶性肿瘤手术患者深静脉血栓形成的预测价值研究
44 淋巴瘤患者化疗后症状集群及生存质量的相关性研究
45 全脑放疗对患者的认知功能及抑郁状态影响研究
46 恶性肿瘤患者心理需求实证研究
47 肺癌患者主要照顾者支持性照护干预方案的构建与应用
48 基于 5A 护理模式的结直肠恶性肿瘤腹腔热灌注化疗护理管理模式构建
49 家庭支持性综合呼吸训练对肺癌手术患者自我效能感及生活质量的影响研究
50 肠造口患者“一网一链”延续性护理管理模式的建立
51 富干细胞脂肪移植在改善乳房假体重建后包膜挛缩的应用研究
52 新型材料自体牙骨粉应用于头颈骨缺损的研究
53 血糖管理系统在肿瘤合并糖尿病患者中的应用
54 经阴道“削苹果式”标本取出法在腹腔镜巨大子宫切除术中的应用及推广
55 基于 ctDNA 痕量浓度检测的乳腺癌疗效评估系统研究
56 基于核酸适配体的乳腺癌快速检测新技术研究
57 共载药物传输系统逆转耐药乳腺癌的机制研究
58 基于肺部 CT 深度流形对齐的 NSCLC 脑转移预测的构建和验证
59 不同孕周内皮功能评估的血流动力学影响机制
60 基于深度学习的恶性肿瘤合并静脉血栓栓塞症风险预测研究
61 新型骨水泥治疗多发性骨髓瘤骨病的实验研究
62 镁合金在食道中上段环境的生物相容性及降解调控
63 基于能谱 CT 技术的前列腺癌识别研究
64 温度-pH-磁三重智能响应微胶囊载药体系在乳腺肿瘤中的靶向输运、定点释放及传质特性研究
65 SIRT1/β-catenin 信号轴在调控结直肠癌 CD8+T 细胞的机制研究

地址：重庆市沙坪坝区汉渝路 181 号 邮编：400030
电话：023-65327159 传真:023-65305442 Email:65305442@163.com

66 AR 技术联合达芬奇机器人在充气式纵隔镜食管癌手术中的应用研究

67 靶向 PARP-1 分子探针研制及其在前列腺癌 PET 显像中的研究

68 基于宽度学习和元学习的阴道分泌物形态学检验诊断研究

69 基于多模态感知和深度迁移学习的中医肿瘤四诊机器人的研究

70 基于动态肺剂量体积参数的放射性肺炎预估模型建立及应用

71 柴胡皂苷 D 活化 PI3KAkt 通路抑制阿霉素心肌损伤的分子机制研究

72 纳秒脉冲诱导三阴乳腺癌细胞免疫原性死亡及消融三阴乳腺癌激发免疫应答的研究

73 荧光金纳米粒修饰微流控芯片用于外泌体富集及释放的研究

74 外周血中循环肿瘤细胞（CTCs）与肝癌热灌注 TACE 相关性研究

75 腓肠内侧动脉穿支皮瓣在修复口腔颌面部缺损的临床研究

76 NRP1 对非小细胞肺癌血管及 PDL1 抑制剂疗效的影响研究

77 β-catenin 乙酰化在调控结直肠癌代谢中的作用及机制研究

78 PHLPP 通过内质网应激调控结肠癌细胞的耐药及作用机制

79 IL-6 通过抑制 miR-455-5p 上调 IGF-1R 促进胰腺癌进展的机制研究

主任委员（签名）______

伦理委员会

（盖章）

2019 年 10 月 23 日

地址：重庆市沙坪坝区汉渝路 181 号　　邮编：400030
电话：023-65327159　　传真:023-65305442　　Email:65305442@163.com
